# MAPbI_3_ Microrods-Based Photo Resistor Switches: Fabrication and Electrical Characterization

**DOI:** 10.3390/ma14164385

**Published:** 2021-08-05

**Authors:** Ehsan Raza, Fakhra Aziz, Arti Mishra, Noora Jabor Al-Thani, Zubair Ahmad

**Affiliations:** 1 Department of Electronics, University of Peshawar, Peshawar 25120, Pakistan; ehsanraza88@yahoo.com; 2 Center for Advanced Materials (CAM), Qatar University, Doha 2713, Qatar; artim2831@gmail.com; 3 Department of Electronics, Jinnah College for Women, University of Peshawar, Peshawar 25120, Pakistan; fakhra69@yahoo.com; 4 Qatar University Young Scientists Center (YSC), Qatar University, Doha 2713, Qatar; n.al-thani@qu.edu.qa

**Keywords:** MAPbI_3_, microrod, photo-resistor

## Abstract

The current work proposed the application of methylammonium lead iodide (MAPbI_3_) perovskite microrods toward photo resistor switches. A metal-semiconductor-metal (MSM) configuration with a structure of silver-MAPbI_3_(rods)-silver (Ag/MAPbI_3_/Ag) based photo-resistor was fabricated. The MAPbI_3_ microrods were prepared by adopting a facile low-temperature solution process, and then an independent MAPbI_3_ microrod was employed to the two-terminal device. The morphological and elemental compositional studies of the fabricated MAPbI_3_ microrods were performed using FESEM and EDS, respectively. The voltage-dependent electrical behavior and electronic conduction mechanisms of the fabricated photo-resistors were studied using current–voltage (I–V) characteristics. Different conduction mechanisms were observed at different voltage ranges in dark and under illumination. In dark conditions, the conduction behavior was dominated by typical trap-controlled charge transport mechanisms within the investigated voltage range. However, under illumination, the carrier transport is dominated by the current photogenerated mechanism. This study could extend the promising application of perovskite microrods in photo-induced resistor switches and beyond.

## 1. Introduction

During the last decade, hybrid organic–inorganic perovskites (HOIPs) have been extensively studied for the emerging perovskite solar cell technology and other optoelectronic applications. Among various types of HOIPs, methylammonium lead iodide (MAPbI_3_) has been commonly employed in artificial synapses, photodetectors, light-emitting diodes (LEDs), field-effect transistors, and photo-resistors beyond the photovoltaics (PV) [1,2]. Besides the use of MAPbI_3_-based thin films in PV applications, other low dimensional perovskite structures, including zero-dimensional (0D) quantum dots (QDs), one dimensional (1D) nanowires (NWs) and nano/microrods, and two dimensional (2D) nanoplates, have been widely employed in optoelectronic applications [3,4,5]. Since perovskite crystals offer better performance relative to thin films in terms of effective light absorption, large surface-to-volume ratio, mechanical flexibility, and better charge separation and conductivity, they have been effectively incorporated in optoelectronic devices [6,7,8]. Among different sub-classes of low-dimensional materials, 1D microrods with a well-defined configuration offer superiority in terms of a 3–4-millimeter length range, confined excitons in one-dimension, effective photon coupling, less heterostructure defects, direct charge carriers transport routes, and simple solution-based technique growth [9,10,11,12]. In addition, a relatively higher and long carrier diffusion length (≈41 µm) is also a beneficial key point in 1D structures [10].

Recent research has shown that I^−^ (0.1–0.6 eV) and MA^+^ (0.5–0.8 eV) have low migration activation energies and several types of point defects in MAPbI_3_ have low formation energies, including vacancies (V_Pb_, V_MA_, V_I_), interstitials (MA_i_, I_i_), and antisubstitutions (Pb_I_, MA_Pb_) [13]. These point defects can cause shallow defect levels and then trap charges at these energy levels, revealing that in HOIPs, a strong potential application of photo-resistors can be predicted. The current flowing across a two-terminal perovskite/semiconductor-based capacitor-like structure is preferentially confined in areas localized at defects [14].

Photo-resistors can be employed in a variety of applications such as fire sensing [15], motion sensing, and radiation detection-based devices [16]. Many reports have been made on the photo-resistor applications using CdS [17], CdSe [18], PbS [19], PbSe [20], and ZnO [21]. In contrast, owing to the advantages of low dimensional perovskite crystals, such as fast photosensitivity over a broad absorption spectrum range, we successfully employed facile solution-grown MAPbI_3_ microrods in the photo-resistors. Usually, MSM capacitor-like devices have been fabricated by sandwiching a thin film of a semiconductor between two metals. However, here, a MAPbI_3_ microrod connected between two metal electrodes has been exploited to form a MSM device.

## 2. Materials and Methods

### 2.1. Preparation of Microrods

**Figure 1:**
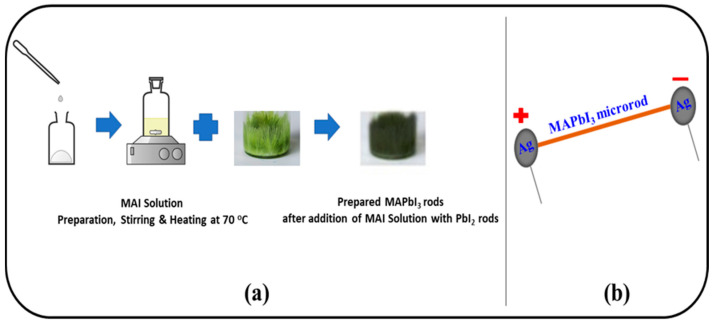
(**a**) Step by step preparation of MAPbI_3_ rods. (**b**) Device Structure of Ag/MAPbI3/Ag-based photo-induced resistor, Reproduced with permission from [12] by Raza et al., Copyright 2020, Springer Nature.

In Dimethylformamide (DMF, anhydrous, 99.8%, Agros chemicals, Broendby, Denmark) and isopropanol (IPA, VWR Prolab chemicals, Laval, QC, Canada), 1 M solutions of each Lead (II) iodide (PbI_2_, Sigma-Aldrich, Darmstadt, Germany) and methylammonium iodide (MAI, TCI Chemicals, Tokyo, Japan), respectively, were prepared separately. To form the solution of MAPbI_3_, already prepared solutions of PbI_2_ and MAI were combined in the volumetric ratio of 2:1. At the initial stage, the thick precipitates of perovskite in the solution were developed. Over time, a crystal-clear yellow solution of the MAPbI_3_ was formed after concurrent heating at 70 °C and stirring at 400 rpm. At a very slow rate of 10 °C/HR, the prepared solution of the MAPbI_3_ was set to cool down to room temperature for 24 h. After passing 24 h, the growth of the microrods was observed. In the vibration-free environment, the length of microrods seems to increase within the solution over time. Step-by-step preparation of MAPbI_3_ microrods is demonstrated in Figure 1a. The microrods were kept for 1000 h in an ambient environment to let them degrade and stabilize after the fabrication of the devices. All the materials were used as received without any purification.

### 2.2. Device Preparation

Commercially available ITO glass substrates (S161, Ossila Ltd, Sheffield, UK) were cleaned as per a well-established cleaning procedure, i.e., ultrasonication in hot DI water mixed with 1% Hellmanex III liquid (for 5 min), followed by rinsing in hot DI water without Hellmanex III liquid. The substrates were then dumped and sonicated in Isopropyl alcohol (for 5 min) and finally rinsed in normal DI water. Later on, the substrates were dried using a stream of dry nitrogen. An individual MAPbI_3_ rod was placed over the pre-patterned ITO electrodes on S161 glass substrates. Then, the silver (Ag) paste was placed at both ends of the rod to form an Ag/MAPbI_3_/Ag-based photo-resistor. We prepared two devices with the diameter of the rods as 50 µm each and lengths of 3.5 and 4 mm (Supplementary Materials Figure S1). The calculated areas are 1.25 and 1.5 µm^2^ for rods with 3.5- and 4-millimeter lengths. The device structure of the Ag/MAPbI_3_/Ag-based photo-resistor is depicted in Figure 1b.

### 2.3. Characterization

To study the surface morphology of the microrods, a field emission scanning electron microscope (FESEM) study was performed. The microrods’ elemental compositional analysis was carried out using Energy-dispersive X-ray spectroscopy (EDS) and X-ray photoelectron spectroscopy (XPS). The monochromatic radiation was used in XPS measurement. In addition, the crystallinity measurement of the microrods was performed using X-ray diffraction (XRD) analysis with a step size of 0.013° of 2θ. Current–voltage (I–V) characterization of the Ag/MAPbI_3_/Ag-based device was measured under dark and 1 Sun illumination (with a scan rate of 10 mV/s) using an Oriel AAA solar simulator (Newport) and Keithley 2400 source measuring unit (SMU). The I–V measurements were conducted under the periodic dc voltage supply considering that one Ag electrode was provided with the voltage while the other was grounded.

## 3. Results and Discussion

**Figure 2:**
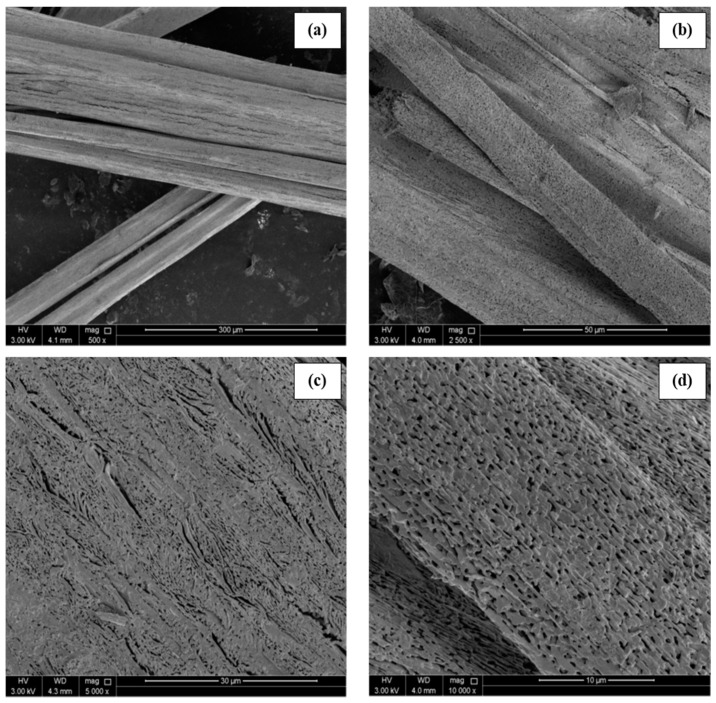
Field emission scanning electron microscope (FESEM) images of the MAPbI_3_ exhibiting porous structure at different magnification levels. (**a**) 300 µm, (**b**) 50 µm, (**c**) 30 µm, and (**d**) 10 µm.

**Figure 3:**
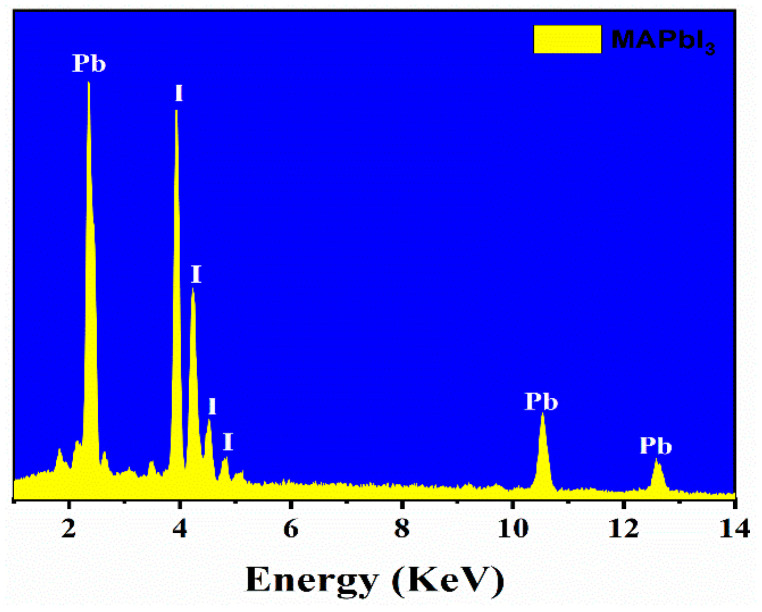
Energy-dispersive X-ray spectroscopy (EDS) spectra of MAPbI_3_-based microrods showing peaks of lead (Pb) and iodine (I) as prominent components.

Figure 2 illustrates the FESEM analysis of MAPbI_3_ microrods under various magnifications. The micrographs reveal the porous structure of MAPbI_3_ microrods. A well-aligned parallel arrangement of the submicron level perovskite crystals in the longitudinal direction is demonstrated. The perovskite microrods display a diameter in the range of ~10–50 μm; however, the length of microrods was up to several millimeters (~3–5 mm), as reported previously [9]. During the growth process, crystals are combined in such a way that hollow regions are created between the adjacent crystals. Nevertheless, no adjacent cracks are observed at the magnification level given in Figure 2. EDS data presented in Figure 3 demonstrate a well-defined stoichiometry of MAPbI_3,_ indicating lead (Pb) and iodine (I) presence as the main ingredients. Two prominent peaks exist at 2.32 and 10.5 keV correlated to Pb, while the peaks at 3.98 and 4.2 keV correspond to the I element in the MAPbI_3_ crystals. Furthermore, the atomic composition of microcrystals reveals the presence of Pb with I in the ratio of 1:2.3, which is consistent with PbI_2_, conforming to the formation of pure phases. In addition, the full EDS data of MAPbI_3_ rods is provided in Supplementary Materials Figure S2. The carbon (C) peak is clearly visible in the full spectrum. The formation of C peak could be attributed to the preparation of MAPbI_3_ rods in DMF solution.

**Figure 4:**
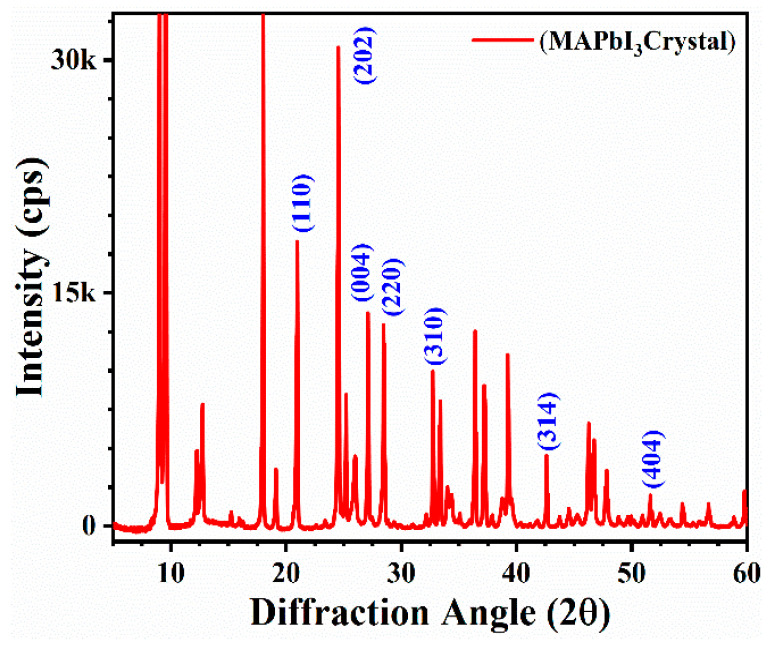
X-ray diffraction (XRD) spectra are showing the crystalline nature of MAPbI_3_-based microrods.

The XRD results are demonstrated in Figure 4. The results showed the hexagonal structure with a P3m1(164) space group for the MAPbI_3_ crystals. The high-intensity diffraction peaks are prominent at low angles, such as 9.0, 9.5, 18.1, and 24.5°, and they strongly dominate the diffraction pattern. The high crystalline nature of the MAPbI_3_ phase formation is verified by the highly intense diffraction peaks assigned to (110), (202), (004), and (220). Additionally, few low-intensity peaks of MAPbI_3_ also exist at (310), (314), and (404) [22]. At room temperature, the unidentified peaks reflecting the stable phase did not match either the pure PbI_2_ or the MAI phase. This implies a new intermediate phase, which could be a PbI_2_–DMF or PbI_2_–MAI–DMF complex [23]. In addition, we have performed XRD analysis for as-synthesis and fresh samples (over 10,000 h). Interestingly, the results demonstrate no significant change. The peaks are prominent in both results. However, the intensities of the peaks have been reduced in fresh samples. These results indicate the crystallinity nature in the MAPbI_3_ rods with no elemental change.

**Figure 5:**
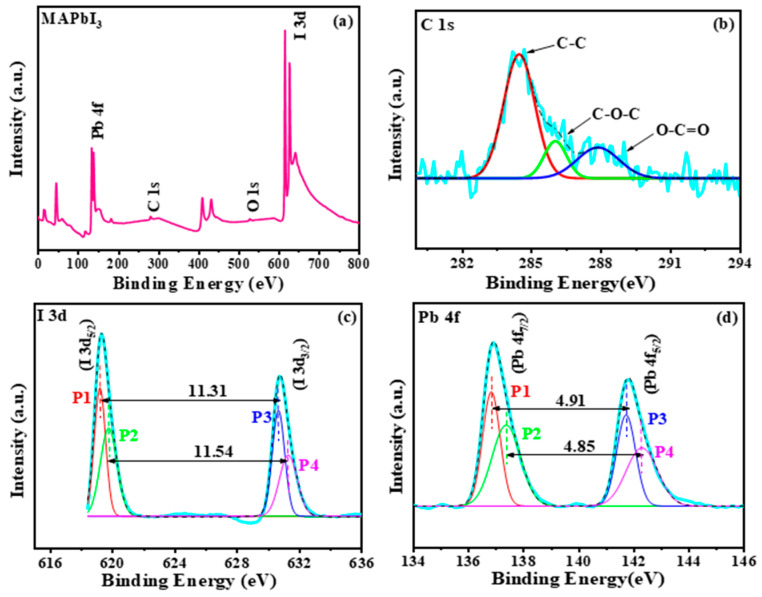
XPS analysis (**a**) survey spectra of MAPbI_3_ crystals, (**b**) core level spectra of carbon (C 1s), (**c**) core level spectra of iodide (I 3d), (**d**) core level spectra of lead (Pb 4f).

The elementary composition analysis of the MAPbI_3_ crystals was performed using XPS. Figure 5a shows the survey spectrum was recorded in the binding energy range between 0 and 800 eV, with the identified elements comprising lead (Pb), iodine (I), oxygen (O), and carbon (C). The existence of O and C shows exposure of crystals to the atmosphere after synthesis. The elemental concentration data are derived from the XPS survey spectrum shown in Table S1. The atomic concentrations of lead (26.95%), iodine (47.57%), and carbon (C) are found as 25.47% in MAPbI_3_ crystals. In addition, the calculated ratio of Pb and I is found as 1:1.765121. This confirms the existence of intermediate phases due to the addition of the MAI.

In addition, XPS core spectra were obtained to explain the changes in crystal growth chemical bonding. To precisely explain features, Shirley background subtraction has been performed before Gaussian curve fitting. The spectra in C 1 s on the surface of the crystals are shown in Figure 5b. The C 1 s spectrum of the MAPbI_3_ crystals shows elements at 284.6, 286.3, and 288.3 eV. This refers to the C-C, C-O-C, and O-C=O bonds, respectively. The core level iodine range has been shown in Figure 5c with two different (P1 and P3) iodine peaks at about 619.20 eV, I(3d_5/2_) and 630.51 eV, I(3d_3/2_) associated with iodine anions from CH_3_NH_3_I and peaks (P2 and P4) at about 619.77 eV, I(3d_5/2_) and 63.31 eV, I(3d_3/2_) for Pb-I bonding. As shown in the core level spectrum of Pb (Figure 5d), four different peaks (P1, P2, P3, P4) have been associated to obtain a perfect fit, the first, P1 at 136.86 eV and the other, P3 at 141.77 eV, represent the Pb(4f_7/2_) and Pb(4f_5/2_) cations associated with PbI_6_. Furthermore, P3 at 137.43 eV and the additional P4 at 142.28 eV represent the Pb^2+^ cation associated with PbO. However, it must be noted that the intensity of P1 and P3 is much more than the P2 and P3. Furthermore, energy separation between peaks P1 and P3 is 4.91 eV, while P2 and P3 maintain 4.85 eV, hence suggesting that crystals mainly have PbI_6_ with strong binding [24,25].

**Figure 6:**
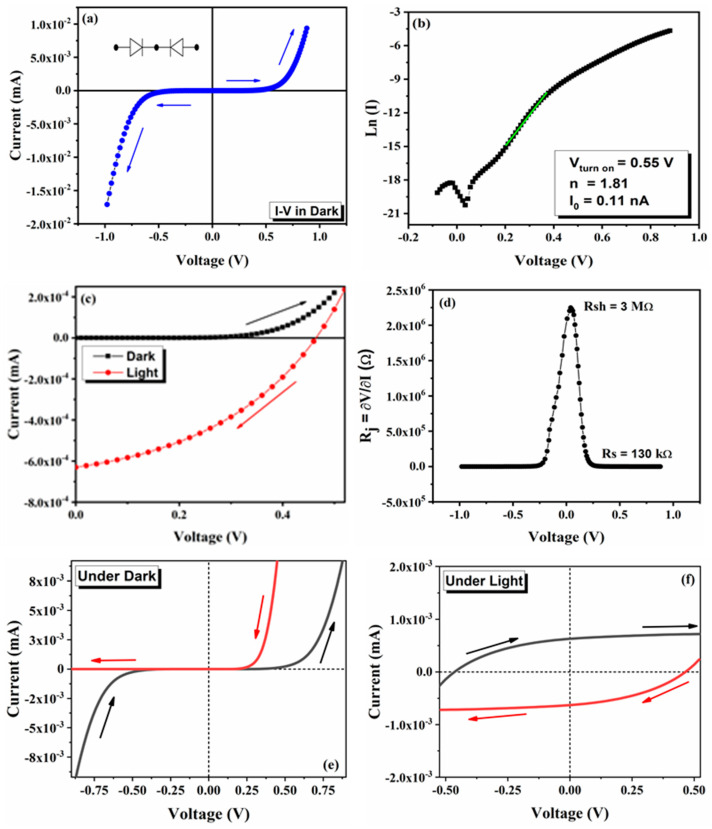
I–V characteristics of fabricated Ag/MAPbI3/Ag-based photo-resistor; (**a**) an initial I–V curve of the device that exhibits a non-linear characteristic, the curves are scanned from 0 to +V and 0 to −V as marked over the curves; (**b**) typical semilog I–V characteristics, the values of average rectified current (Io), ideality factor (n) are mentioned in the inset; (**c**) I–V characteristics in the dark and under light (one sun illumination); and (**d**) Junction resistance (Rj) versus voltage (V) showing series and shunt resistances as R_s_ and R_sh_, respectively. Arrows indicate the scanning directions. I–V hysteresis curves of Ag/MAPbI_3_/Ag-based photo-resistor (**e**) under dark and (**f**) under light conditions. Arrows indicate the scanning directions (−V to 0).

The I–V characteristics of the Ag/MAPbI_3_/Ag-based photo-resistor are recorded in the voltage ranging from −1 to +1 V, as shown in Figure 6a. The MAPbI_3_ microrod attached with the Ag contacts exhibit nonlinear features and demonstrates metal/semiconductor/metal (M/S/M) junction behavior. The typical I–V characteristics of the Ag/MAPbI_3_/Ag-based photo-resistor on the semi-log scale are plotted in Figure 6b. As shown by the red curve in Figure 6c, the photovoltaic current is generated under illumination. The turn-on voltage was found to be 0.55 V. The series and shunt resistances, R_s_ and R_sh_, resistances are shown in Figure 6d. The values obtained for the R_s_ and R_sh_ are 130 kΩ and 3 MΩ, respectively. For the fabricated photo-resistor, the value of the ideality factor “n” was calculated as 1.85 ± 0.4. The ideality factor for an ideal diode must be close to one, but in most of the metal/semiconductor interfaces, this value is deviated from unity and is observed to be greater than one. This larger value implies secondary conduction mechanisms at the interface. The I–V curves under dark and light conditions with scan directions (−V to 0) for an Ag/MAPbI_3_/Ag-based photo-resistor are demonstrated in Figure 6e,f, respectively. A significant hysteresis effect can be observed in both cases.

**Figure 7:**
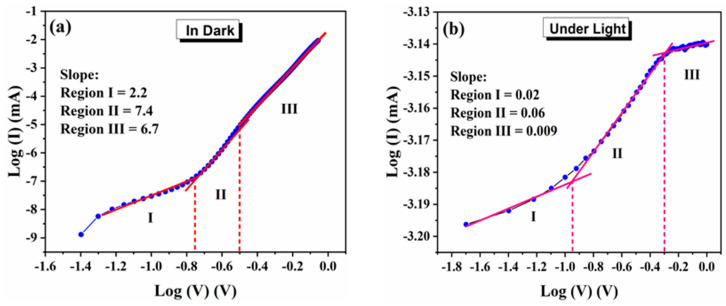
Log V–Log I plots for Ag/MAPbI_3_/Ag-based photo-resistor (**a**) under dark and (**b**) under illumination (100 mW/cm^2^).

To study the possible conduction mechanism in the dark and under light, the I–V characteristics of microrod-based Ag/MAPbI_3_/Ag photo-resistor are plotted in a log V–log I scale as shown in Figure 7a,b, respectively. There are three major regions with distinct slopes, namely, the region I, II, and III, observed in Figure 7a. The slope of the fitting line in region I (between −1.4 and −0.75 V) is estimated as 2.2 (close to 2) that obeys I ∝ V^2^ (child’s law), demonstrating a space charge limited current (SCLC) transport mechanism. In regions II and III, the slope takes the values of 7.4 and 6.3, respectively, indicating a trapped charge limited current (TCLC) in a voltage range greater than 0.75 V. Among the proposed models, the SCLC and the TCLC mechanism can be employed to elucidate the conduction mechanism of our device under dark. Region I is dominated by the SCLC conduction mechanism in which the injected electrons participate in the current flow and govern the conduction mechanism. The trapping of injected carriers by the inherent defects in the perovskite layer (MAPbI_3_) causes the current to flow proportional to the applied bias square. The electrons and traps in the perovskite layer play an essential role in the charge transport mechanism. It has been broadly argued that the SCLC is strongly correlated to the switching layer’s charge trapping and dielectric quality. In Figure 7b, there are three distinct regions, e.g., regions I, II, and III. Region I demonstrates a slope of 0.02, region II shows a slope of 0.06, and Region III represents a slope of 0.009. Typically, the slope with a value of one represents the ohmic state. However, in our case, all the slopes are <1 and close to 0. This indicates that the density of the photogenerated carriers dominates the electrical transport within the Ag/MAPbI_3_/Ag device. In region I, the photogenerated carrier concentration exceeds the injected carriers, while in region II, a rapid increase in the current is observed. This could be attributed to the fact that few trapped electrons absorbed the incident photons and became excited [26]. Moreover, it can be assumed that the charged ions trap some of the photo-excited carriers at the perovskite interface, reducing the current flow in region III. The accumulated charges are beneficial for the application of hybrid organic–inorganic perovskites-based photo-resistors [27].

**Figure 8:**
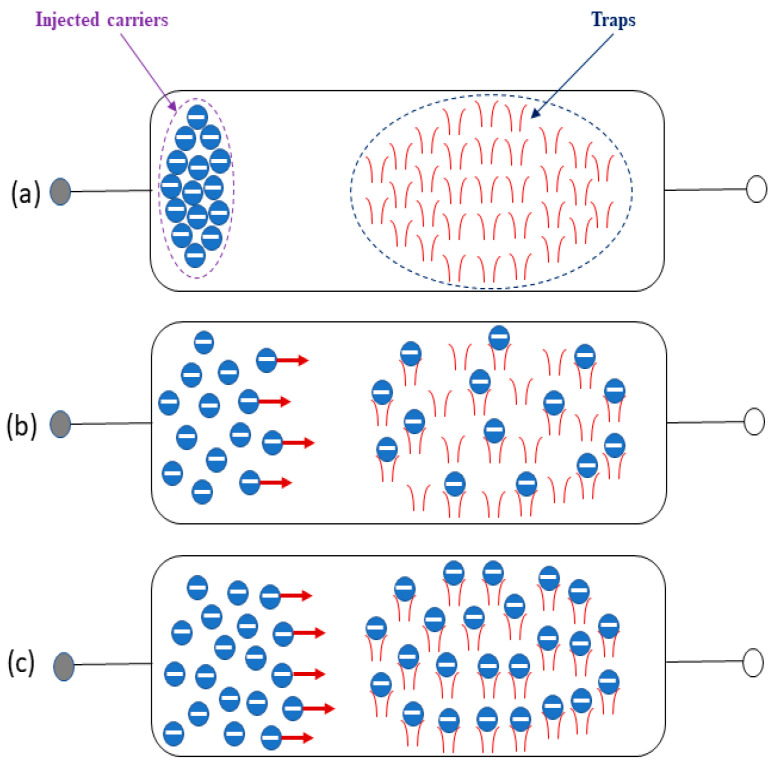
Distribution of carriers in MAPbI_3_-based microrods under strong carrier injection in space-charge-limited conduction. (**a**) Thoroughly trapped behavior, (**b**) partially filled traps (trapped behavior), (**c**) Strong injection, complete traps filled up (traps filled up (traps free behavior)) Reproduced with permission from [28] by F. C. Chiu.

A possible elucidation of the transport mechanism in the Ag/MAPbI_3_/Ag photo resistor could be the presence of interface traps generated due to vacancies and interstitials defects of MAPbI_3_. Inherently, when the potential is applied at the Ag electrode, the injected carriers rapidly increase at the interface and become entrapped by the traps at the Ag-MAPbI_3_ interface induced by the intrinsic defects of MAPbI_3_. The trap filling up process is demonstrated in Figure 8. These filled traps provide a smooth pathway for the conduction of electrons leading to the increased current value. Accordingly, the entrapped carriers (electrons in our case) in this region account for limiting the current flow.

**Figure 9:**
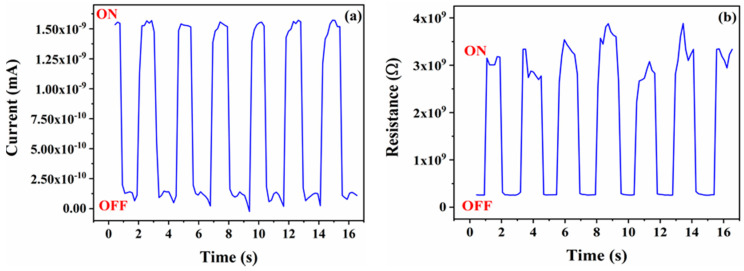
Photo-sensitivity vs time response of Ag/MAPbI_3_/Ag-based photo-resistor demonstrating (**a**) high and low currents and (**b**) high and low resistances.

The photo-sensitivity characteristics of the Ag/MAPbI_3_/Ag-based device were performed, whereas noticeable differences in high and low currents and resistances are presented in Figure 9a,b, respectively. Moreover, under an illumination intensity of 100 mWcm^−2^ with an external bias voltage of 0.4 V, the device was set to a low resistance state (LRS), and during the absence of light illumination, the device exhibited a high resistance state (HRS). The applied initial electric field was weakened by the reverse electric field caused by space charges; this prevents the injection of charge carriers. Therefore, the device changes from ON to OFF. The light illumination intensity releases the trapped space charges, which disappear due to the internal electric field; hence, it switches OFF state to ON.

A comparison of the photosensitivity of an MAPbI_3_-based thin film and a 1D microrod is presented in Table 1. Here, the results of the 1D microrod indicate a superior performance in terms of photosensitivity compared to the MAPbI_3_-based thin films and even with nanowires. The photosensitivity (*S*) is defined in (1) as follows [29,30]:

(1) $$S=\frac{I_{p}}{P_{in}}$$

where *I_p_* and *P_in_* represent photo-current and incident light power, respectively. The *I_p_* is further defined as $I_{p}=I_{light}-I_{dark}$.

## 4. Conclusions

In summary, we have demonstrated the possible application of MAPbI_3_ microrods-based Ag/MAPbI_3_/Ag devices for photo-resistors. FESEM images exhibit the porous structure of microrods. An EDS study demonstrates the Pb and I as main components. In addition, the structural analysis was carried out using XRD, and the results revealed the pure formation of MAPbI_3_ microrods. Different conduction mechanisms were observed at different voltage ranges in the dark and under illumination. In dark conditions, the conduction behavior was dominated by typical trap-controlled charge transport mechanisms within the investigated voltage range. However, under illumination, the carrier transport is dominated by the current photogenerated mechanism. The device parameters such as average rectified current (0.11 nA), ideality factor (1.4), and barrier height (0.6 eV) were extracted using the current–voltage (I–V) characteristics of the photo-resistor. The obtained values for the R_s_ and R_sh_ are found as 130 kΩ and 3 MΩ, respectively. The results show that the perovskite microrods can potentially fabricate sensitive and fast photoelectric photo-resistor devices.

## Figures and Tables

**Table 1:** Comparison of photosensitivity of MAPbI_3_-based thin films and 1D microrod.

Device Structure	Perovskite Formation	Photosensitivity (A.W^−1^)	Bias Voltage (V)	Reference
Au/MAPbI_3_/Au	Film	0.0111	5	[31]
Au/MAPbI_3_/Au	Film	0.408	5	[32]
Au/MAPbI_3_/Au	Nanowires	0.055	0.1	[33]
Ag/MAPbI_3_/Ag	Microrods	0.76	0.4	Present Work

## Data Availability

No data available.

## Supplementary Materials

The following are available online at https://www.mdpi.com/article/10.3390/ma14164385/s1, Figure S1: The image of the developed MAPbI_3_ based device on ITO substrates. The image is zoomed-in and shown in right side of the image, Figure S2: Energy-dispersive X-ray spectroscopy (EDS) spectra of MAPbI3 based micro-rods showing peaks of lead (Pb) & iodine (I) as prominent components. The carbon (C) peak is also visible at low voltage values, Figure S3: XRD analysis of as-synthesis and fresh crystals of MAPbI_3_ based micro-rods, Figure S4: XPS analysis survey spectra of MAPbI_3_ crystals with full binding energy (0–1200 eV) and without any background subtraction and baseline correction, Table S1: Elemental composition data extracted from XPS analysis.
