# Supplementary material for: MAPbI3 Microrods-Based Photo Resistor Switches: Fabrication and Electrical Characterization
Source: Materials (Basel). 2021 Aug 5;14(16):4385. doi: 10.3390/ma14164385 (PMC8398296; doi:10.3390/ma14164385)
Supplement: Supplementary file 1 [file materials-14-04385-s001.zip › materials-1248780-supplementary.pdf]

materials

MDPI

Supplementary Materials

$MAPbI_3$ Microrods-Based Photo Resistor Switches: Fabrication and Electrical Characterization

Ehsan Raza [1,2], Fakhra Aziz [3], Arti Mishra [2], Noora Jabor Al-Thani [4] and Zubair Ahmad [2,4,*]

[1] Department of Electronics, University of Peshawar, Peshawar 25120, Pakistan; ehsanraza88@yahoo.com
[2] Center for Advanced Materials (CAM), Qatar University, Doha 2713, Qatar; artim2831@gmail.com
[3] Department of Electronics, Jinnah College for Women, University of Peshawar, Peshawar 25120, Pakistan; fakhra69@yahoo.com
[4] Qatar University Young Scientists Center (YSC), Qatar University, Doha 2713, Qatar; n.al-thani@qu.edu.qa
* Correspondence: zubairtarar@qu.edu.qa; Tel.: +974-4403-7729

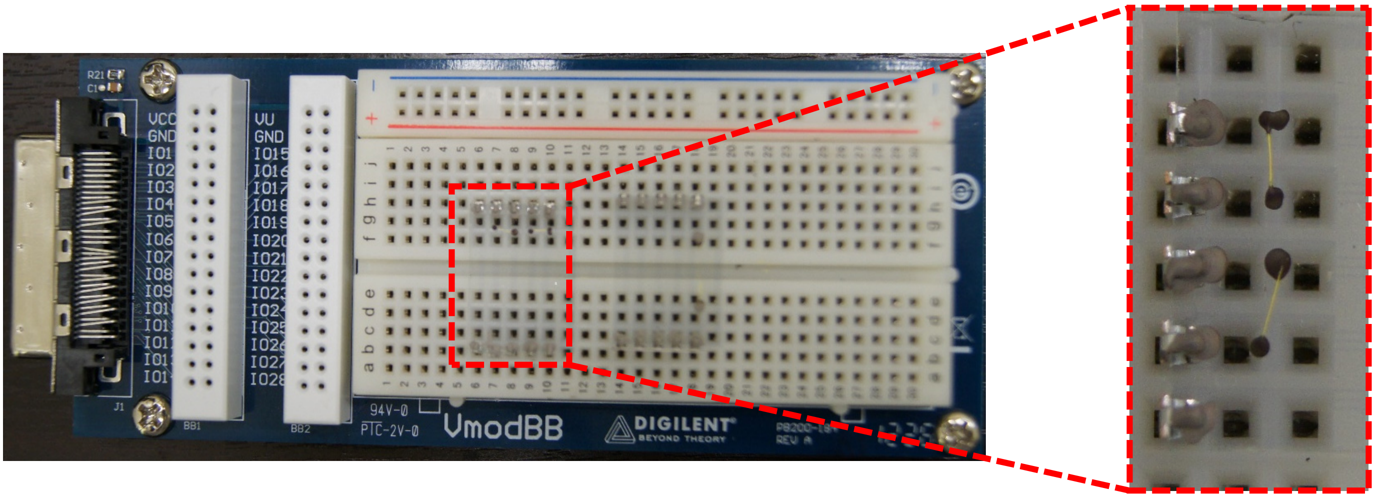

Figure S1. The image of the developed $MAPbI_3$ based device on ITO substrates. The image is zoomed-in and shown in right side of the image.

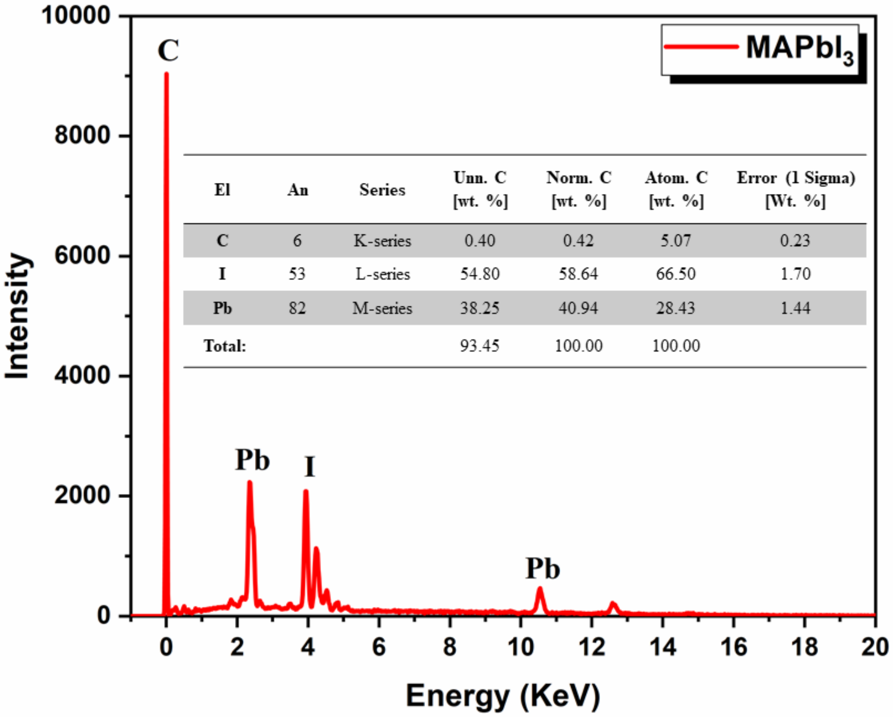

El	An	Series	Unn. C [wt. %]	Norm. C [wt. %]	Atom. C [wt. %]	Error (1 Sigma) [Wt. %]
C	6	K-series	0.40	0.42	5.07	0.23
I	53	L-series	54.80	58.64	66.50	1.70
Pb	82	M-series	38.25	40.94	28.43	1.44
Total:			93.45	100.00	100.00	

Figure S2. Energy-dispersive X-ray spectroscopy (EDS) spectra of $MAPbI_3$ based micro-rods showing peaks of lead (Pb) & iodine (I) as prominent components. The carbon (C) peak is also visible at low voltage values.

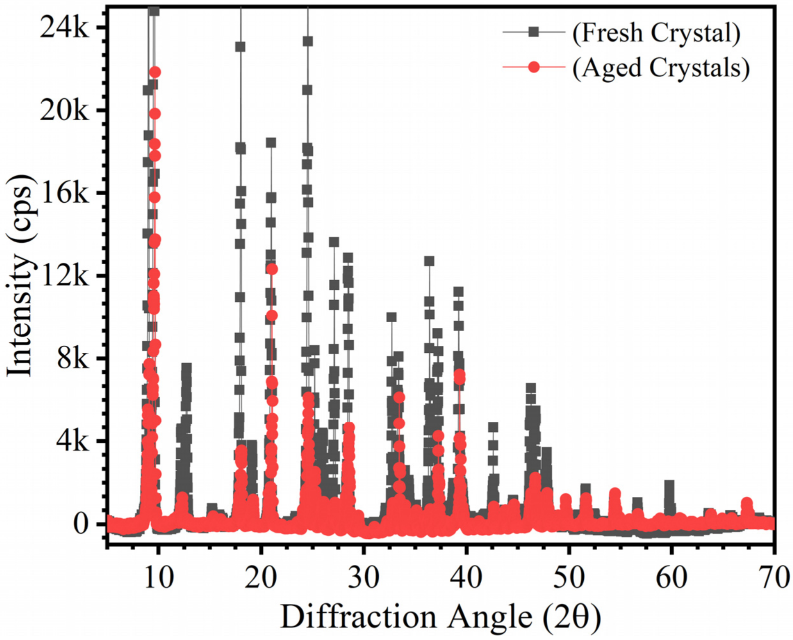

Figure S3. XRD analysis of as-synthesis and fresh crystals of $MAPbI_3$ based micro-rods.

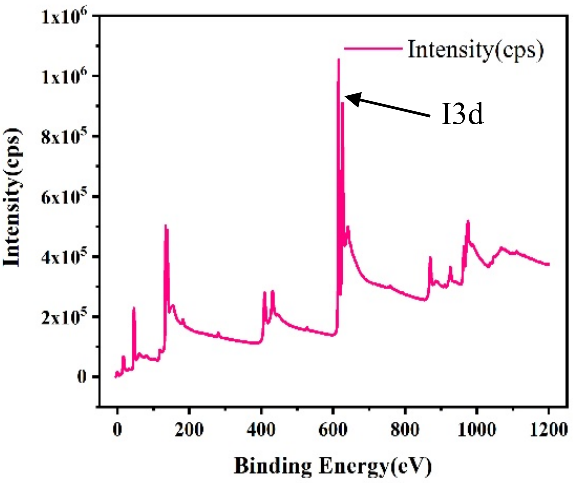

Figure S4. XPS analysis survey spectra of $MAPbI_3$ crystals with full binding energy (0-1200 eV) and without any background subtraction and baseline correction.

Table S1. Elemental composition data extracted from XPS analysis.

El	Atomic Concentration (%)	Mass Concentration (%)
**C**	25.47	2.57
**Pb**	26.95	46.82
**I**	47.57	50.61
